# Techno-Economic Assessment of Integrated Wastewater Technologies for Sustainable Treatment of Highly Loaded Landfill Leachate Using GPS-X^TM^

**DOI:** 10.3390/bioengineering13030359

**Published:** 2026-03-19

**Authors:** Abdulmohsen Abdulkarim Mohammed Alkunaydiri, Nuhu Dalhat Mu’azu, Ahmad Hussaini Jagaba

**Affiliations:** 1 Department of Environmental Engineering, College of Engineering, Imam Abdulrahman Bin Faisal University, Dammam 31451, Saudi Arabia; 2240500189@iau.edu.sa; 2 Department of Civil Engineering, Faculty of Engineering, University of Tabuk, P.O. Box 741, Tabuk 71491, Saudi Arabia; ajagaba@ut.edu.sa

**Keywords:** landfill leachate treatment, biological treatment processes, wastewater treatment simulation, water treatment modeling, membrane bioreactor (MBR), municipal solid waste landfill, advanced wastewater treatment technologies, water treatment OPEX and CAPEX, net present value (NPV) and life cycle cost (LCC)

## Abstract

Landfill leachate is considered one of the most recalcitrant wastewaters due to its high organic strength, elevated ammonia concentrations, and complex chemical composition. This study evaluates integrated technologies for treating highly loaded landfill leachate from the Wadi Al-Asla landfill, Jeddah Saudi Arabia, using GPS-X^TM^ modeling combined with regulatory compliance and techno-economic assessment (TEA). The characterized mature leachate exhibited extremely high average concentrations of COD (17,050 mg L^−1^), BOD_5_ (10,058 mg L^−1^), ammonia-N (989 mg L^−1^), and total nitrogen (1223 mg L^−1^), indicating severe pollution levels requiring integrated treatment technologies. Five (5) different scenarios involving integrated biological, physicochemical, and membrane-based processes were modelled, simulated and evaluated against local discharge standards complaince. Conventional and municipality-proposed upgrade configurations achieved ~80–83% COD removal, producing effluent COD > 2900 mg L^−1^ and 1790–1801 mg L^−1^ BOD_5_, indicating persistent non-compliance for organic pollutants. Nitrogen removal improved substantially (93.7–95.7% ammonia-N and 91–93% total nitrogen removal), yet residual ammonia-N (44–63 mg L^−1^) and total nitrogen (92–108 mg L^−1^) remained above regulatory limits. Advanced hybrid systems achieved complete TSS removal and strong phosphorus control (TP ≤ 0.42 mg L^−1^), while three(3) compartmental aerobic–anoxic membrane bioreactor coupled with reverse osmosis (MBR + RO) achieved near-complete nitrogen removal and reduced 90% COD removal. The lifecyle economic assessment indicated OPEX ranging from USD 1.1 to 5.6 m^−3^ of treated leachate with the aerobic–anoxic MBR + RO configuration yieding footprint advantage, lower CAPEX and moderate OPEX By combining process modeling, regulatory compliance evaluation, and economic assessment, this study provides a practical screening framework for selecting sustainable treatment strategies for high-strength landfill leachate and wastewater matices.

## 1. Introduction

Municipal solid waste management (MSWM) has become one of the most critical environmental challenges worldwide due to rapid urbanization, population growth, and industrial development. Landfilling remains the most adopted method for final waste disposal, particularly in developing countries, due to its relatively low cost and operational simplicity [1]. However, one of the major environmental issues associated with landfills is the generation of leachate; a highly contaminated liquid that forms when precipitation or surface water infiltrates through the waste layers, dissolving and mobilizing pollutants [1,2,3].

The composition and quantity of landfill leachate depend on several factors, including the age of the landfill, the type of waste deposited, local climatic conditions, and the degree of biological degradation occurring within the landfill cells [4]. Typically, landfill leachate contains organic matter (expressed as BOD and COD), ammonia nitrogen, heavy metals, chlorides, sulfates, and various xenobiotic organic compounds. The leachate can migrate into the subsurface and contaminate soil and groundwater, posing long-term ecological and public health risks.

Effective leachate management thus plays a vital role in minimizing the environmental footprint of landfills. Treatment methods generally involve a combination of physical, chemical, and biological processes such as aeration, coagulation-flocculation, membrane filtration, and biological nitrification–denitrification [2]. Current global policy targets a selection of technologies that would ensure a more close-looped wastewater treatment system for achieving sustainable and cost-effective treatment systems [5]. The selection and optimization of treatment processes depend on the leachate characteristics, flow rate, and regulatory discharge limits. In this context, process simulation tools such as GPS-X^TM^ have become increasingly important, allowing engineers and researchers to model treatment systems, evaluate process interactions, and optimize operational conditions without the need for extensive field experimentation. Modeling is crucial for effective treatment and understanding and mitigating the environmental impacts of wastewater as well as landfill leachates [6,7], particularly concerning groundwater contamination. Various approaches have been employed, including numerical models, artificial neural networks, and integrated mathematical frameworks. For instance, a one-dimensional numerical model developed by Pramada and Anjana quantifies contaminant transport in aquifers, highlighting the significance of landfill design on groundwater quality [8]. Javad and Saeedi utilized a neural network to predict leachate generation based on meteorological data, achieving a high correlation with measured data, thus demonstrating the effectiveness of machine learning in this context [9]. Additionally, Mulkey et al. explored initial conditions for modeling leachate migration, revealing that different modeling scenarios can significantly affect predicted groundwater concentrations [10]. Mannina et al. [11] proposed an integrated model to simulate leachate fluxes and gas production, emphasizing the importance of understanding both organic and inorganic contaminant dynamics within landfills [12]. Badruddin’s study on the Tamangapa landfill employed Modflow and MT3DMS to simulate leachate dispersion and develop a remediation plan, illustrating practical applications of modeling in real-world scenarios [13]. Collectively, these studies underscore the complexity of leachate behavior and the necessity for robust modeling techniques to inform landfill management and environmental protection strategies.

Wastewater treatment plant (WWTP) modeling using GPS-X has become an important tool for evaluating, optimizing, and managing the environmental performance of treatment systems handling complex waste streams such as landfill leachate [14]. GPS-X provides a mechanistic, mass-balance-based simulation platform capable of representing key biochemical and physicochemical processes, including activated sludge kinetics, nitrification–denitrification, biological phosphorus removal, and solids separation. As a result, the model has been widely applied to assess the treatability of high-strength leachate and its potential environmental implications, particularly when linked to pollutant loading, process limitations, and compliance performance [15]. In addition, several studies have demonstrated the capability of GPS-X to simulate a wide range of wastewater unit operations and integrated treatment trains, supporting WWTP capacity assessment, scenario comparison, process troubleshooting, and optimization of operational parameters such as HRT, SRT, aeration demand, and membrane performance [16]. Furthermore, numerical models developed for leachate migration highlight the importance of accurately quantifying contaminant transport in aquifers, emphasizing the need for robust modeling approaches to predict leachate behavior and its potential groundwater contamination [8,17]. These insights underscore the necessity of integrating advanced modeling techniques like GPS-X in leachate management strategies to mitigate environmental risks.

The WAL is a landfill that has faced operational challenges related to leachate accumulation and treatment inefficiency. The leachate from this landfill poses a serious environmental concern due to its high levels of pollutants like ammonia, COD, BOD, heavy metals, and salts, making treatment difficult. This increases risks such as groundwater contamination, soil degradation, and odor emissions, highlighting the urgent need for effective treatment to meet NCEC regulatory reuse or discharge requirement. Understanding the characteristics of the generated leachate, assessing the current treatment system, and exploring optimized operational scenarios through simulation are essential steps toward achieving sustainable landfill management and preventing future environmental contamination.

Although numerous studies have investigated landfill leachate treatment technologies and performance optimization, most of the existing research has focused on general treatment processes or laboratory-scale evaluations rather than site-specific operational modeling [1,3]. Moreover, recent comprehensive reviews of landfill leachate treatment technologies highlight that mature leachate presents complex mixtures of organic, nitrogenous, and emerging contaminants that often exceed the removal capacity of conventional methods, and emphasize the importance of integrated and innovative treatment designs, including membrane processes and advanced oxidation techniques, to achieve regulatory compliance and sustainability goals [18].

In this context, limited studies have addressed the actual performance of engineered landfills and the efficiency of their leachate treatment systems under real operational conditions. At the WAL, while an integrated leachate management system was originally implemented, its long-term performance, treatment efficiency, and operational resilience have not been systematically analyzed. The WAL leachate treatment plant revealed weaknesses in the existing system’s capacity, maintenance, and adaptability to fluctuating waste inputs and climatic variations.

The novelty of this work lies in the systematic comparison of multiple integrated landfill leachate treatment configurations within a single, unified modeling framework using a consistent influent dataset representative of mature, high-strength leachate. Unlike many previous studies that focus on individual treatment technologies or laboratory-scale evaluations for leachate treatment, this study simultaneously evaluates conventional biological systems, municipal upgrade configurations, anaerobic membrane bioreactors (AnMBR), advanced polishing options, and hybrid membrane bioreactor–reverse osmosis (MBR + RO) systems under identical operating conditions using GPS-X^TM^ process modeling. This approach enables a direct comparison of treatment performance, regulatory compliance, sludge management implications, and techno-economic feasibility across alternative treatment pathways. Furthermore, the study integrates effluent quality assessment, regulatory compliance analysis, and lifecycle techno-economic evaluation to identify a practical and scalable treatment strategy for extremely high-strength landfill leachate. By linking process modeling outcomes with operational and economic considerations, the work provides decision-support insights for upgrading the existing treatment infrastructure at the Wadi Al-Asla landfill. The proposed framework also offers a transferable methodology for evaluating and optimizing sustainable leachate treatment strategies in other high-load or resource-constrained landfill systems, thereby contributing to improved management of complex wastewater streams.

## 2. Materials and Methods

### 2.1. The Study Aea: Wadi Al-Asla Landfill

**Figure 1:**
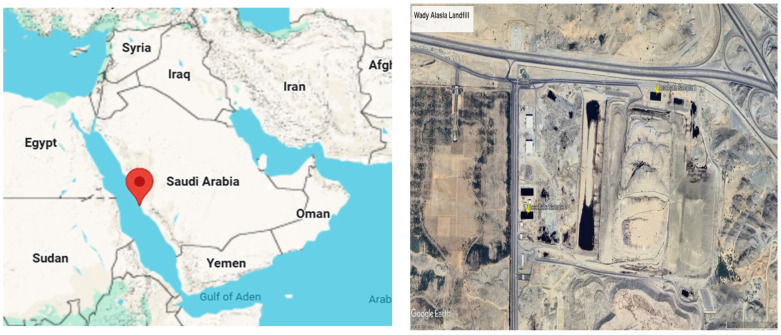
The study area.

**Figure 2:**
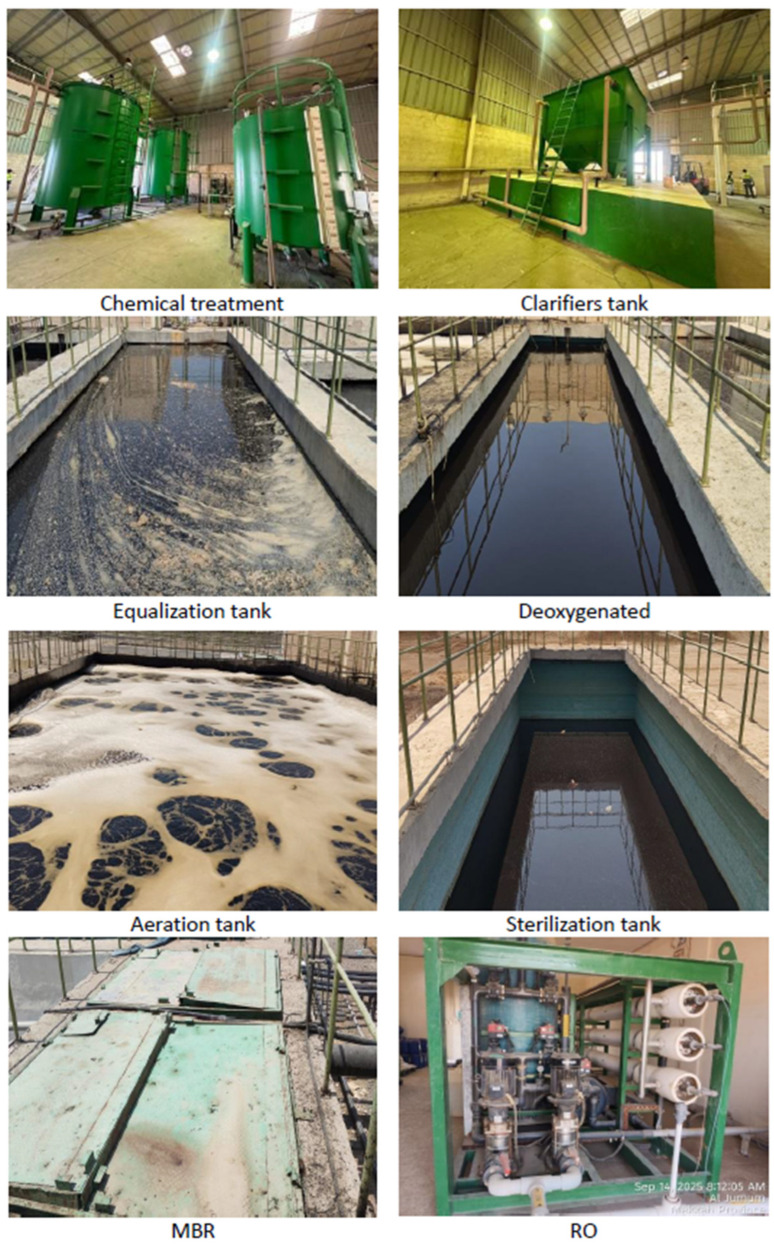
Leachate treatment plant in Wadi Al-Asla landfill during shutdown period.

The study area (Figure 1) is the Wadi Al-Asla Landfill (WAL), located east of Jeddah in the Makkah Province of the Kingdom of Saudi Arabia, approximately 35–40 km from the Jeddah city center. The landfill is situated within the Wadi Al-Asla, a seasonal valley system that originates in the inland eastern highlands and drains westward toward the Red Sea. Geographically, the site is located at approximately 21.644194° N latitude and 39.390260° E longitude, with an average elevation of about 170 m above mean sea level. During its initial years of operation, the landfill functioned efficiently due to the integration of a waste sorting facility that reduced the volume of organic and recyclable materials entering the disposal cells. This upstream waste management approach contributed to lower leachate generation rates and supported overall environmental performance. However, following the shutdown of the sorting facility, the landfill system experienced a decline in operational performance as shown in Figure 2. Prolonged accumulation of untreated leachate resulted in hydraulic overloading, blockages within the leachate collection network, and localized seepage from landfill cells into the surrounding environment, highlighting the need for an improved and robust leachate management strategy.

### 2.2. Data Collection Procedure

Landfill leachate samples were collected from the WAL, east of Jeddah, Saudi Arabia. Sampling locations were selected following a reconnaissance survey to ensure representative site conditions, and their geographic coordinates were recorded using a Global Positioning System (GPS). Samples were collected in pre-cleaned, sterilized high-density polyethylene (HDPE) bottles, rinsed three times with leachate prior to collection. Sample preservation and handling followed Standard Methods for the Examination of Water and Wastewater (APHA, 2017), including acidification with nitric acid for heavy metal analyses. Samples were stored at approximately 4 °C and transported to the Environmental Measurement Company Limited (EMCO) laboratory in Jeddah for analysis within recommended holding times. Quality assurance and quality control (QA/QC) procedures included field blanks, laboratory blanks, duplicate sample analyses, and instrument calibration using certified reference standards. Calibration verification was performed at regular intervals, and analytical precision was confirmed through duplicate measurements. Chain-of-custody documentation was maintained throughout sampling, transport, and analysis to ensure data integrity and traceability.

### 2.3. GPS-X Modeling Setup

Among many other WWTP modeling softwares, GPS-X^TM^ version 9.1 the latest version of a comprehensive plant-wide modeling platform from Hydromantis Environmental Software Solutions Inc. Mississauga, ON, Canada, ref. [19] has gained significant attention in recent years [14,15,20]. Its popularity stems from an extensive library of precompiled treatment technologies, user-friendly interface, robust support for IWA ASM variants (alongside advanced models like Mantis), powerful tools for sensitivity analysis, optimization, and dynamic simulations, plus readily available training resources and documentation [19]. This makes it particularly valuable for engineers tackling complex municipal and industrial wastewater scenarios. Thus, a model of the existing leachate treatment plant was developed using GPS-X software. The model replicates the treatment processes including primary settling, biological treatment, aeration, and chemical dosing in terms of the flowrates and the dimensions of the various units. Considering the complexity of handling different scenarios as well as the divergent of the treatment units involved, the GPS-X^TM^ default parameter values were adopted, which were found to be adequate for the default scenario calibration, corroborating previous study [15].

### 2.4. Technological Scenarios and Performance Evaluation

Selecting appropriate technologies for municipal solid waste (MSW) landfill leachate treatment remains challenging due to the complex and highly variable nature of leachate composition [1]. Leachate characteristics evolve with landfill age, climate, and waste composition, ranging from biodegradable organics in young leachate to refractory compounds, ammonia, heavy metals, and emerging contaminants in mature leachate. Evidently, from a number of recent reiews [3], this variability complicates the process design and often necessitates site-specific solutions rather than standardized approaches. Increasingly stringent discharge regulations, limited land availability, and the need for long-term operational reliability further influence technology selection. As a result, effective leachate management typically requires the integration of multiple complementary treatment technologies to address diverse contaminants while balancing technical performance, economic feasibility, and environmental sustainability [1,2,3].

**Figure 3:**
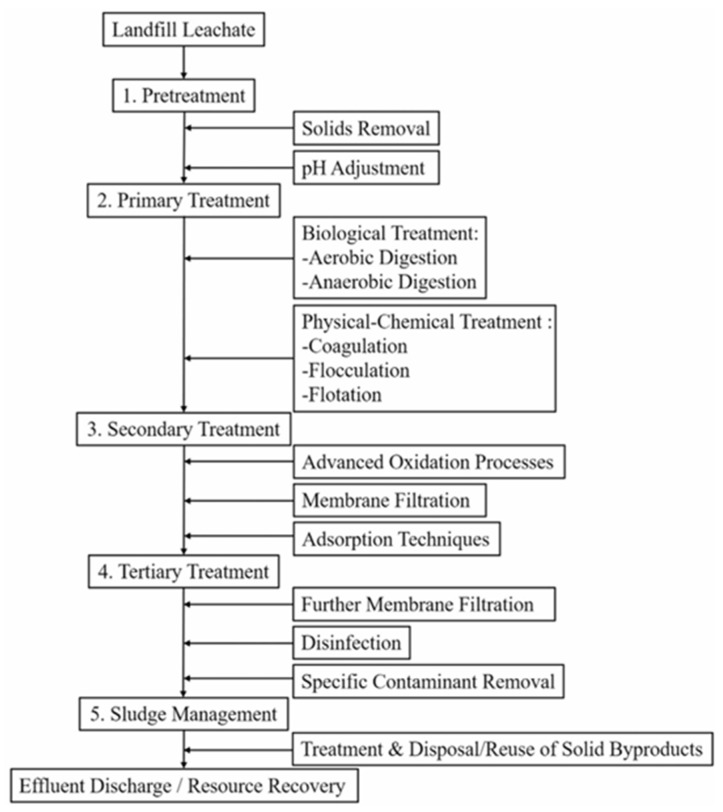
Combination of technologies for MSW landfill leachate [1].

In the present study, the five (5) scenarios of combinations of physicochemical and biological treatment technologies which are in line with the generic approach in Figure are considered [1]. As provided in Table 1, these combined treatment approaches included Scenario 1 which serves as the baseline which represents the existing treatment system installed at WAL (designated as Scenario 1). The unit processes as provided in Figure 3 consist of coagulation flocculation, primary clarifier, equalization tanks, anoxic tank, AnMBR, disinfection, and RO units. Meanwhile, two (2) upgraded proposals are provided by Jeddah Municipality (Scenarios 2 and 3) in the WAL design master plan. As well, Scenarios 4 and 5 as proposed improvement were considered. For Scenario 4, two clarifiers were introduced to Scenario 1, placed after anoxic and activated sludge aerobic tanks, to provide clarification prior to the downstream treatment processes. For the 5th scenario, a three-compartment MBR was opted for to serve both the need for anoxic and aerobic processes along with their effluent clarification during the leachate treatment process, thus eliminating the need for the conventional tanks and clarifiers to explore lesser footprints due to coupled, more advanced technological alternatives. Simulation results were analyzed to assess the effectiveness of the treatment plant under the tested scenarios. Performance indicators included pollutant removal efficiency (BOD, COD, etc.), compliance with NCEC for discharge standards, and potential reduction of environmental impacts.

### 2.5. Modeling, Simulation and Data Analysis

To address the challenges identified at the WAL, this research adopts an integrated analytical and modeling approach to assess and optimize the performance of the leachate treatment system. The methodology of this project involves a systematic approach to simulate and evaluate the performance of the existing leachate treatment plant at WAL using GPS-X^TM^ software version 9.1. The development plan consists of the following steps:

Step-1. Data Collection and Characterization: Gather operational data from the landfill and analyze the physicochemical properties of the leachate, including concentrations of organic matter, heavy metals, salts, and toxic compounds.
Step-2. Model Setup: Develop a dynamic simulation model in GPS-X^TM^ to replicate the processes of the existing treatment plant.
Step-3. Scenario Analysis: Simulate different operational scenarios, including variations in flow rate, pollutant load, and treatment configurations, to assess their impact on treatment efficiency and environmental compliance.
Step-4. Performance Evaluation: Analyze simulation results to determine the most efficient operational conditions, including pollutant removal efficiency, compliance with local standards, and potential environmental impact reduction.
Step-5. Recommendations and Development Plan: Provide guidance for optimizing future operations of the treatment plant and inform decision making for similar landfill leachate management projects.

### 2.6. Economic Feasibility Assessments

#### 2.6.1. CAPEX and OPEX Analysis

Comparative techno-economic assessment for relative economic feasibility of the five (5) investigated scenarios was performed at a screening level for a treatment capacity of 700 m^3^ d^−1^ considering the capital and operational expenditures (CAPEX and OPEX). The CAPEX estimation incorporated modeled reactor volumes, hydraulic capacities, equipment sizing, and the total number and complexity of unit operations within each treatment configuration. OPEX evaluation considered energy consumption, chemical dosing requirements, sludge handling, routine maintenance, and component replacement costs. The cost estimation was performed using literature-reported cost coefficients as given in Table S1. The unit CAPEX and OPEX cost estimation ranges adopted given in Table S1 were based on different reliable literature sources, US EPA, WEF, etc. [21,22,23,24,25,26,27,28,29]. Accordingly, the unit cost coefficients were adopted for high-strength landfill leachate treatment systems from literature-reported ranges for the biological, physicochemical, and advanced treatment systems, typically expressed per unit treatment capacity (USD per m^3^·day) or per unit treated volume (USD per m^3^). This methodology enables consistent system-wide comparison and provides a rational basis for evaluating lifecycle cost intensity and relative economic feasibility across the alternative scenarios.

#### 2.6.2. Lifecycle Cost (LCC) and Net Present Value (NPV) Analysis

To complement the compliance-driven technical evaluation, a life cycle cost (LCC) framework was adopted to compare the economic burden of the five integrated treatment scenarios over a defined project horizon. LCC represents the present value of total expenditures required to construct and operate each treatment configuration, including initial capital investment and recurring annual operating costs:

(1) $$\mathrm{LCC}=\mathrm{CAPEX}+\sum_{t=1}^{N}\frac{{OPEX}_{t}}{{\left(1+r\right)}^{t}}$$

where CAPEX is the installed capital cost (USD), OPEX*_t_* is the annual operating cost in year *t* (USD·yr^−1^), *r* is the discount rate (%), and *N* is the project lifetime (years). Annual operating cost was expressed as

(2) OPEXt=OPEX×Qannual

(3) Q_annual_ = Q_d_ × 365

where OPEX*_m_*_3_ is the operating cost per treated volume (USD m^−3^), Q is the annual treated volume (m^3^·yr^−1^), and Q*_d_* is the daily treated flow (m^3^·d^−1^). Using the LCC framework, the net present value (NPV) of each scenario was computed to quantify the discounted lifecycle economic burden over a selected 20-year operational period.

## 3. Results

### 3.1. WAL Leachate Characteristics

The average characteristics of leachate samples from Jeddah WAL are provided in Table 2. A comparison with recently reported international data highlights its relatively severe pollution load. The WAL leachate exhibited a very high concentration of ammonia (NH_3_–N) (≈989 mg/L), total nitrogen (≈1223 mg/L) which is comparable to or exceeds values reported for mature leachates in India, Turkey, China, and other countries across the globe [3,30]. Moreover, the COD (≈17,050 mg/L) and BOD (≈10,058 mg/L) values are substantially higher than many of those recently reported in MSW leachate studies, indicating a strong organic load and partial biodegradability [18]. The biodegradability index of the characterized leachate was evaluated using the BOD_5_/COD ratio, which provides an indication of the biodegradable organic fraction. Based on the measured average concentrations, the resulting BOD_5_/COD ratio of approximately 0.59 indicates the presence of a significant biodegradable organic fraction. Although mature landfill leachate is typically associated with lower BOD_5_/COD ratios (<0.3), the elevated ratio observed in the WAL leachate suggests mixed-age leachate characteristics, likely influenced by ongoing waste deposition and possible mixing of leachate from younger waste layers. Such conditions can produce leachate with simultaneously high ammonia concentrations and relatively biodegradable organic content, requiring integrated biological and advanced highly advanced treatment technologies.

### 3.2. GPS-X Model Calibration, Validation and Scenarios Modeling

#### 3.2.1. GPS-X MANTIS Modeling Framework

In this study, the process simulations were performed using GPS-X^TM^ version 9.1, a wastewater treatment modeling software, developed by Hydromantis Environmental Software Solutions. GPS-X^TM^ is a widely used dynamic simulation platform for modeling biological, physicochemical, and membrane-based wastewater treatment processes [19]. The biological treatment units were implemented using the MANTIS2-activated sludge model library (MANTIS2LIB), which represents an extended formulation derived from Activated Sludge Model No.1 (ASM1). The MANTIS2 model incorporates additional microbial growth pathways for heterotrophic and autotrophic organisms and enables simulation of nitrification, denitrification, and simultaneous nitrogen transformation processes.

The implemented model contains more than 60 state and composite variables, governed by over 30 stoichiometric and 24 kinetic parameters describing microbial growth, substrate utilization, oxygen consumption, and nitrogen conversion. For the present WAL leachate simulations, the treatment system was configured using the carbon–nitrogen (CN) process library of the MANTIS2 ASP model together with the Simple1D secondary clarifier model. This configuration allows simulation of carbon degradation, nitrification, and denitrification pathways, enabling prediction of the removal of key pollutants including COD, BOD_5_, ammoniacal nitrogen, and total nitrogen across the evaluated treatment scenarios.

#### 3.2.2. GPS-X Model Calibration and Validation

The calibration results indicate that the GPS-X^TM^ model reproduced several key effluent parameters with good agreement compared to the measured data as provided in Table 3. In particular, COD showed excellent prediction, with a predicted value of 4641 mg L^−1^ compared to the measured 4700 mg L^−1^, corresponding to a deviation of less than 2%. The model also accurately simulated pH and dissolved oxygen, suggesting that aeration dynamics, oxygen transfer, and general system conditions were well represented. Reasonable agreement was also observed for total phosphorus, indicating that phosphorus behavior in the treatment system was adequately captured. Although BOD was somewhat overestimated (2846 vs. 2350 mg L^−1^), the predicted value remains within the same order of magnitude, suggesting that the biodegradable organic fraction and heterotrophic activity were generally well represented in the calibrated model.

Greater deviations were observed for nitrogen-related parameters, particularly NH_3_–N and TN, where the model predicted lower concentrations than those measured in the effluent, indicating an apparent overestimation of nitrification performance. However, part of this discrepancy can be attributed to uncertainties in the original measured nitrogen data, which required adjustment of the influent nitrogen fractions in the GPS-X^TM^ model to ensure stoichiometric consistency between state variables and composite nitrogen components within the model framework. Such adjustments are often necessary to maintain mass balance closure and proper representation of nitrogen species (organic N, ammonia, nitrate) within the model structure. Consequently, while some differences remain in the nitrogen predictions, the calibration generally captures the the treatment dynamics and organic matter removal, and is considered sufficiently robust for subsequent scenario evaluation and comparative analysis.

Due to limited availability of measured effluent data for two representative leachate samples obtained from the WAL, the validation step focused on comparing the GPS-X^TM^-predicted effluent concentrations with the corresponding influent characteristics of these two samples, and the results were assessed relative to the calibration performance to evaluate the consistency and robustness of the model predictions. The validation results demonstrate that the predicted effluent concentrations for major parameters follow trends similar to those observed during calibration, particularly for COD, BOD, pH, and dissolved oxygen, indicating that the calibrated model maintains stable predictive behavior when applied to independent influent datasets.

Similarly, eventhough greater variations are observed in nitrogen-related parameters, the overall prediction patterns remain consistent with the calibration results, reflecting similar limitations associated with nitrogen fractionation and the complex composition of mature landfill leachate. Despite the restricted validation dataset, the comparison between validation outputs and calibration performance confirms that the model predictions remain within the same order of magnitude and follow the expected treatment behavior, suggesting that the calibrated GPS-X^TM^ model provides a reasonable and internally consistent representation of the treatment system. Therefore, the GPS-X^TM^ model default parameters’ validation supports the reliability of the calibrated model for comparative scenario analysis and performance evaluation of alternative treatment configurations investigated.

#### 3.2.3. Scenarios GPS-X^TM^ Modeling

**Figure 4:**
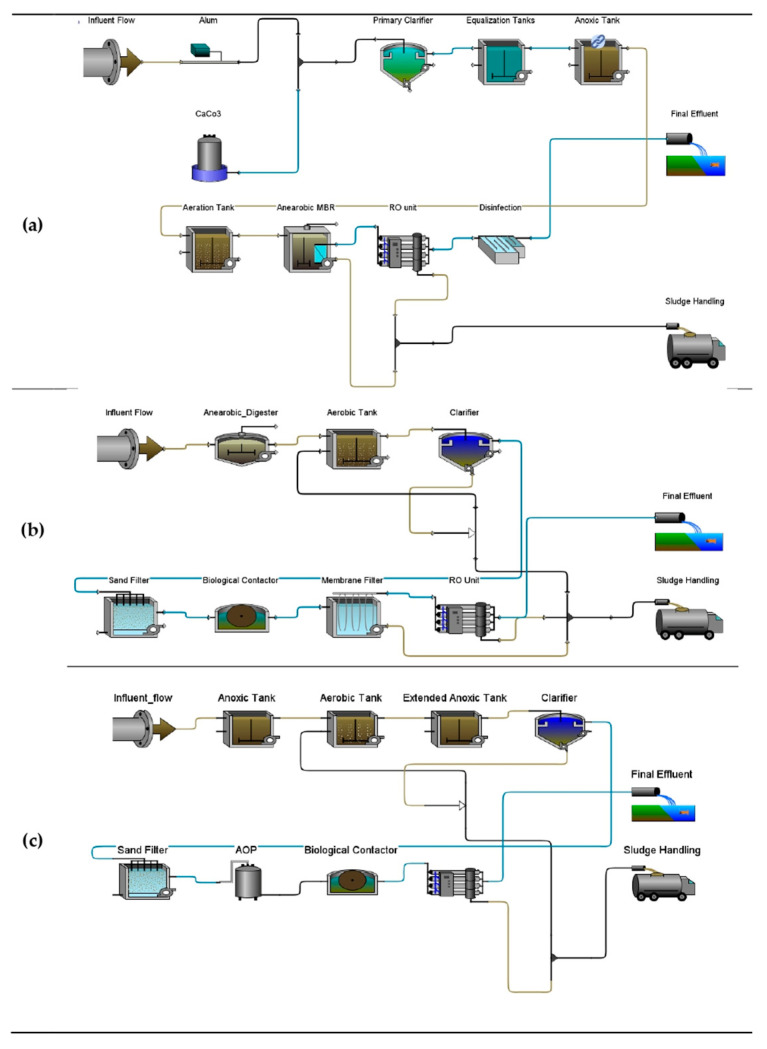
GPS-X^TM^ modeling layouts for (**a**) existing plant Scenario 1 and the WAL master plan upgrades (**b**) Scenario 2 and (**c**) Scenario 3.

**Figure 5:**
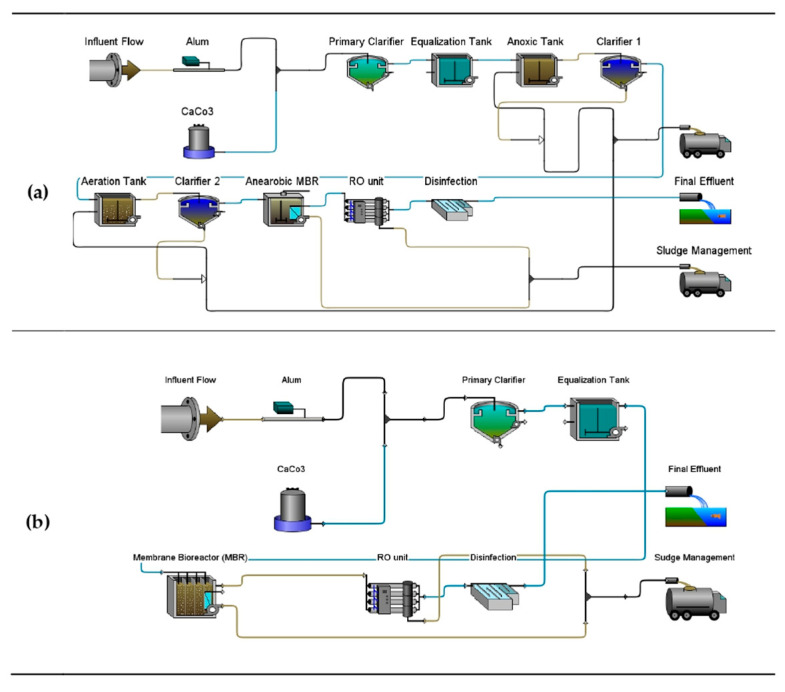
GPS-X^TM^ modeling layouts for proposed plant improvement (**a**) Scenario 4 and (**b**) Scenario 5.

Figure 4 and Figure 5 provide the five layouts represented in the GPS-X^TM^ software Version 9.1 modeling environment which were used to simulate five (5) scenarios for the WAL Leachate Treatment Plant. The objective of the simulations was to evaluate the performance of the existing plant configuration (Scenario 1) and compare it with four proposed upgrade scenarios (Scenarios 2–5), with the aim of identifying the most efficient and sustainable treatment strategy for achieving regulatory compliance and improving effluent quality. Each scenario is assessed based on pollutant removal efficiency, compliance with environmental discharge limits, unit-process performance, and, where applicable, economic indicators.

#### 3.2.4. Performance of WAL Baseline and Plant Master Plan Upgrades Scenarios

**Figure 6:**
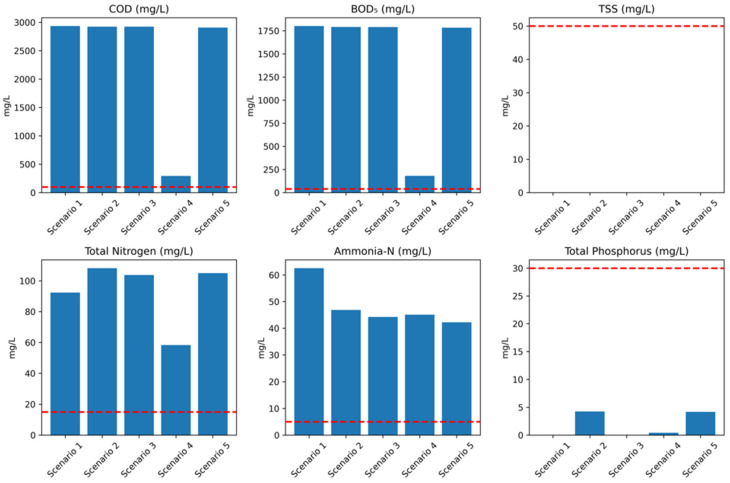
Comparison of GPS-X^TM^ simulation of effluent quality parameters for the different scenarios with red dotted line indicating regulatory limits.

The simulation results for the five (5) treatment configurations modeled using GPS-X^TM^ are summarized in Table 4. Meanwhile, Figure 6 illustrates complaince with the local quality discharge regulation thresholds for the various parameters. The existing treatment plant (Scenario 1) operates with an influent flow rate of 700 m^3^ d^−1^ and produces approximately 573.75 m^3^ d^−1^ of treated effluent. Table 4 presents the influent and final effluent concentrations together with the corresponding removal efficiencies for each scenario. The GPS-X^TM^ simulation confirms the existing operational condition represented by Scenario 1, demonstrating that despite relatively high removal efficiencies—particularly for total suspended solids (TSSs) and total phosphorus (TP)—the treated effluent concentrations remain significantly above regulatory discharge limits. The biological treatment stage exhibits insufficient removal of organic matter and nitrogen, indicating limitations associated with inadequate reactor capacity and/or insufficient hydraulic and solids retention times. Consequently, the reverse osmosis (RO) unit receives excessively high pollutant loads, which reduces its effectiveness as a polishing step. Thus, Scenario 1 fails to meet discharge standards for all major organic and nitrogenous parameters.

The first master plan option proposed by Jeddah Municipality for treatment improvement (Scenario 2) incorporates several process modifications intended to enhance the treatment train effectiveness and performance. However, the simulated results indicate that the final effluent continues to exceed regulatory discharge limits for nearly all key parameters. Persistent non-compliance is observed for most pollutants, with regulatory conformity achieved only for total suspended solids (TSSs) and total phosphorus (as soluble phosphate). The principal exceedances occur for BOD_5_, total nitrogen, and ammoniacal nitrogen, reflecting the high-strength characteristics of the influent leachate. The reverse osmosis (RO) unit operates at a recovery rate of 60%, treating a feed flow of 676 m^3^ d^−1^ and producing 405.6 m^3^ d^−1^ of permeate and 270.4 m^3^ d^−1^ of concentrate. The elevated influent pollutant loads are identified as the primary limiting factor constraining the RO unit’s ability to consistently achieve full regulatory compliance.

On the other hand, the second treatment improvement option proposed by Jeddah Municipality (Scenario 3) represents a substantial enhancement in the technological treatment performance compared with the existing configuration and the first upgrade option. The simulation results indicate high technical effectiveness in pollutant removal; however, the system does not consistently achieve full compliance with all NCEC discharge limits. Scenario 3 achieves complete removal of TSSs and TP, with final effluent concentrations of 0.00 mg L^−1^ for both parameters. This performance is primarily attributed to the integration of sand filtration followed by RO, which provides effective solids separation and phosphorus removal. The RO unit operates at an approximate water recovery of 60%, producing 411.6 m^3^ d^−1^ of treated permeate from the incoming feed. Despite these improvements, exceedances persist for selected organic and nitrogenous parameters, indicating that additional process intensification or polishing would be required to ensure full regulatory compliance.

Meanwhile, with respect to organic matter and nutrient removal, the AOP contributes significantly to leachate pretreatment; however, the final effluent concentrations of COD and BOD_5_ remain elevated at 2923.57 mg L^−1^ and 1790.63 mg L^−1^, respectively. Although the system demonstrates strong nitrogen removal performance, achieving reductions of 95.5% for ammoniacal nitrogen and 91.7% for total nitrogen, the residual concentrations (44.23 mg L^−1^ for ammonia-N and 103.78 mg L^−1^ for total nitrogen) still exceed the NCEC discharge limits of 5 mg L^−1^ and 15 mg L^−1^, respectively. Consequently, while there is no violation for solids and phosphorus, the system remains non-compliant for organic and nitrogenous parameters. The AOP unit was implemented using the GPS-X^TM^ oxidation module, where the main operational parameters include the oxidant dose (in mg L^−1^) and the oxidant effectiveness factor, which represents the fraction of the applied oxidant effectively contributing to pollutant oxidation. For this study, the GPS-X^TM^ default values for these two parameters were adopted following calibration and validation of the treatment model. For that, these results indicate that further optimization of AOP operating conditions and/or the incorporation of additional biological or physicochemical polishing stages is required to achieve full regulatory compliance.

#### 3.2.5. Performance of Proposed Modifications Scenarios

The proposed modification scenarios (Scenarios 4 and 5) represent targeted upgrades to the existing treatment configuration (Scenario 1), as outlined in Table 1 and Section 2.4. Scenario 4, illustrated by the GPS-X^TM^ process layout in Figure 5a, incorporates an AnMBR and demonstrates enhanced treatment capability alongside notable sustainability benefits. Simulation results indicate that, although substantial performance improvements are achieved, the final effluent does not fully comply with all NCEC discharge limits. Specifically, residual concentrations of COD (293.5 mg L^−1^), BOD_5_ (180.6 mg L^−1^), and ammonia-N (45.2 mg L^−1^) remain above regulatory thresholds, resulting in non-compliance for these parameters. In contrast, full compliance is achieved for TSSs and TP, both of which are reduced to near-zero concentrations

The unit process performance highlights confirm the operational robustness of Scenario 4. The AnMBR effectively treats high-strength leachate while offering the potential for energy recovery through biogas production under the default GPS-X^TM^ parametrization, which would require optimization to fully harness its economic potential. In addition, the RO unit operates at a water recovery of approximately 80%, producing about 340 m^3^ d^−1^ of treated permeate. While Scenario 4 provides clear sustainability advantages compared with earlier scenarios, further optimization of biological treatment intensity and/or the addition of downstream polishing steps is required to achieve full regulatory compliance across all monitored pollutants.

In contrast, Scenario 5 (process layout shown in Figure 5b) represents the present study second recommended improvement configuration and offers the most balanced overall performance among the evaluated options. This scenario yielded an excellent treatment efficiency with high removal rates across key parameters and presents a favorable cost-to-performance ratio, attributable to the reduced number of operational units and a smaller system footprint. Full regulatory compliance is achieved for TSSs, total nitrogen, and total phosphorus; however, residual concentrations of COD, BOD_5_ and ammoniacal nitrogen remain above the stringent NCEC discharge limits. Despite these limited exceedances, Scenario 5 demonstrates superior operational reliability, economic feasibility, and process simplicity compared with the other configurations. When coupled with targeted polishing steps for residual ammonia and biodegradable organics, Scenario 5 emerges as the most practical and scalable treatment option, offering an optimal balance between high treatment efficiency, and reduced process complexity.

The RO unit features in all the investigated scenarios configurations implemented for its relevance as a polishing step following upstream physicochemical and biological treatment units to achieve final effluent quality consistent with regulatory discharge limits. The RO process was represented using a simplified membrane separation model based on the solution–diffusion relationship in Equation (4).

(4) J_w_ = A(ΔP − Δπ)

where Jw is the water flux through the membrane, A is the membrane permeability coefficient, ΔP is the applied pressure, and Δπ is the osmotic pressure difference between the feed and permeate streams.

The estimated osmotic pressure of the feed–brine streams obtained from the GPS-X^TM^ simulation outputs approximately ranged from 5.54 to 9.34 atm and 6.17 to 14.7 atm for the log-mean osmotic pressure and arithmetic mean osmotic pressure, respectively. Moreover, a GPS-X^TM^default solute rejection coefficient of 0.95 was adopted to represent the separation efficiency of thin-film composite polyamide membranes commonly used in leachate treatment systems to be inconsistent with the model calibration and validation. Because increasing recovery leads to progressive concentration of dissolved solutes in the brine stream, which raises osmotic pressure and reduces the net driving pressure for permeation, the recovery ratio was conservatively limited to 60–80% across the five scenarios. Thus, this recovery range balances permeate production with operational stability by limiting osmotic pressure buildup, scaling potential, and membrane fouling under high-salinity leachate conditions.

## 4. Economic Assessments of WAL Leachate Treatment Scenarios

Besides the compliance-driven technical evaluation using GPS-X^TM^ modeling, this study conducted a comparative techno-economic assessment for relative economic feasibility of the five (5) investigated scenarios. The analysis was performed at a screening level for a treatment capacity of 700 m^3^ d^−1^, based on modeled flowrates, typical energy consumption values for biological, advanced oxidation process and membrane systems under the different scenarios, and cost coefficients reported in recent studies. The CAPEX was estimated using unit-capacity cost ranges for anaerobic digesters, aeration tanks, MBR systems, AOP reactors, and RO units, while OPEX included energy demand (kWh m^−3^), chemical consumption (coagulants, oxidants), sludge handling, membrane replacement, and routine maintenance with ranges as provided in Table S1 [21,22,23,24,25,26,27,28,29]. An overall relative ranking was assigned to each scenario based on combined consideration of economic efficiency (lowest cost) and spatial footprint, with rank 1 representing the most favorable option and rank 5 indicating the least favorable configuration.

### 4.1. Scenarios CAPEX Analysis

Considering the economic assessment results in Table 5, it is clear that the CAPEX required to construct a 700 m^3^/day landfill leachate for WAL varies significantly across the five evaluated scenarios, reflecting differences in process complexity, equipment intensity, and footprint requirements. The CAPEX for the various scenario estimates is in the range of 9–32 million with the average of the maximum CAPEX at USD 25.1 million, matching the USD 24 million cost of installing the WAL leachate treatment system. Amongst the scenarios, Scenario 5 exhibits the lowest CAPEX (USD 9–20 million), which can be attributed to its compact and higher integrated advanced technologies design. The MBR eliminates the need for large secondary clarifiers and sand filters, reducing concrete volume and civil works by approximately 40–60% compared with conventional activated-sludge configurations. Scenario 2 follows closely (USD 11–22 million), benefiting from the relatively low-cost anaerobic digester for high-strength leachate pretreatment while still incorporating conventional biological polishing and RO with potential of energy recovery. In contrast, Scenario 3 records the highest CAPEX (USD 16–32 million) because of the additional AOP reactors (ozone/UV or Fenton), associated chemical dosing systems, and larger aeration volumes required. Scenarios 1 and 4 occupy intermediate positions (USD 13–27 million and USD 15–27 million, respectively), driven by the inclusion of multiple biological stages (anoxic tanks, activated-sludge basins, and clarifiers) alongside MBR and RO units.

### 4.2. Scenarios OPEX Analysis

The OPEX constitutes the dominant component of the long-term economic burden, especially for the investigated small-scale landfill leachate treatment plants (700 m^3^/day or 255,500 m^3^/year), often accounting for 60–80% of the economic indices. At this low capacity, the OPEX is elevated on a per-m^3^ basis compared to larger installations due to reduced economies of scale, higher relative fixed costs (e.g., labor, monitoring, and preventive maintenance), and less efficient energy utilization. The estimated OPEX ranges from 1.1–5.6 USD/m^3^ across scenarios (midpoint values 1.3–2.35 USD/m^3^), yielding an annual OPEX of approximately 0.52–0.90 million USD/year (Table 6).

The primary OPEX drivers—energy, chemicals, maintenance (including sludge management), and membrane-related costs—exhibit clear scenario-specific patterns. Energy consumption dominates in membrane-intensive and advanced oxidation processes (AOP), typically 40–70% of total OPEX. Scenario 5 (chemical pretreatment + MBR + RO + disinfection) achieves the lowest midpoint OPEX (1.4 USD/m^3^) which can be attributed to the efficient integrated compact design, yet energy use remains moderate (1.2–3.0 kWh/m^3^), translating to 0.6–1.4 USD/m^3^ at regional electricity tariffs (0.05–0.10 USD/kWh in Saudi Arabia. Maintenance, including membrane cleaning/replacement and sludge dewatering/disposal, adds 0.4–1.1 USD/m^3^, while lowering disposal costs compared to conventional activated sludge.

Scenario 2 (anaerobic digester + conventional biological + RO) follows closely (midpoint ≈ 1.35 USD/m^3^), benefiting from significantly lower energy demands (0.8–2.5 kWh/m^3^, net lower with biogas offsets of 20–50% equivalent) and reduced sludge volume after digestion. Biogas recovery can generate on-site power or heat, lowering demand from the national grid energy price. This renders Scenario 2, competitive as evidenced by the NPV/LCC sensitivity to discount rates and lower exposure to OPEX fluctuations as discussed in the next section.

In contrast, Scenario 3 (biological + AOP + RO) incurs the highest OPEX, driven by intensive energy processes (3.0–10 kWh/m^3^) and chemical reagent costs (0.3–1.3 USD/m^3^). This pushes the annual OPEX to (1.9–5.6 USD/m^3^), which is prone to amplifying the LCC sensitivity to both discount rates and OPEX volatility, especially for a small-scale plant like the present investigated WAL plant. Thus, the elevated cost of this scenario is primarily driven by potential AOP chemical consumption and units with higher associated energy demand, confirming that process intensification significantly increases recurring expenditure

Meanwhile, Scenarios 1 and 4 are at the intermediary preferred options with a midpoint cost of 1.7 and 1.6 USD/m^3^, reflecting the added energy and sludge handling from extended biological stages (anoxic/activated sludge + clarifiers) alongside MBR/RO units. Sludge management-dewatering to 20–30% solids followed by landfill disposal or stabilization typically contributes 10–30% of OPEX (0.3–1.0+ USD/m^3^), higher in aerobic system configurations due to greater biomass yield.

### 4.3. Sensitivity and Uncertainty Considerations

**Figure 7:**
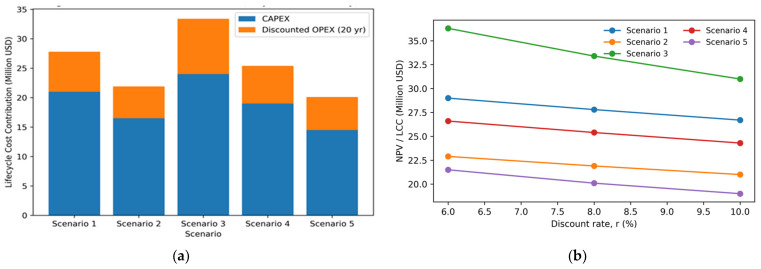

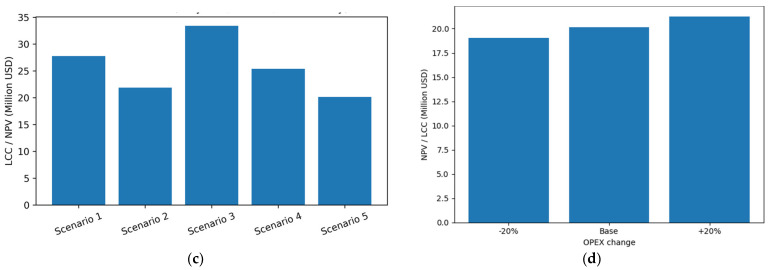
Techno-economic comparison of the five leachate treatment scenarios: (**a**) CAPEX vs. discounted OPEX contributions, (**b**) NPV sensitivity to discount rate, (**c**) lifecycle cost (LCC/NPV) comparison at 8%, and (**d**) Scenario 5 OPEX sensitivity analysis.

To evaluate the robustness of the lifecycle economic ranking under reasonable market and financial uncertainty, a screening-level sensitivity assessment was performed by varying key cost drivers that most strongly influence leachate treatment economics. Specifically, the discount rate was varied across a representative planning range (6–10%), and energy and chemical unit prices were perturbed by ±20% to reflect typical volatility in utility tariffs and reagent supply. These CAPEX values directly translate into the net present value (NPV)/lifecycle cost (LCC) comparison presented in Figure 7a (20-year horizon, 8% discount rate). The bar heights illustrate that, despite Scenario 3 having the highest absolute CAPEX, its elevated OPEX (driven by AOP energy and reagents) amplifies the discounted total cost to ~USD 33.4 million. Conversely, Scenarios 5 and 2 achieve the lowest NPV/LCC (USD 20.1 million and USD 21.9 million, respectively), confirming that lower upfront investment combined with moderate OPEX yields the most favorable economics at the WAL plant scale.

The sensitivity analysis (Figure 7b) further demonstrates that higher discount rates (10%) reduce the relative impact of future OPEX, narrowing the gap between scenarios. Across the induced perturbations, the relative ranking remained stable: Scenario 3 consistently exhibited the highest NPV as results of elevated chemical and energy intensity, while Scenarios 4 and 5 remained the most economically competitive among the advanced treatment configurations. The sensitivity analysis shows that a ±20% variation in operating expenditure results in only a modest change in lifecycle cost for Scenario 5, ranging from approximately USD 19.0 to 21.3 million. This limited variation indicates that the proposed configuration maintains strong economic robustness against fluctuations in energy and chemical prices. The low sensitivity to energy and chemical price fluctuations was a result of the integrated compact configurations that reduces unit redundancy and avoids the high reagent dependency. These findings indicate that the recommended Scenario 5 solution is not only compliance-oriented but also the most economically viable and operationally robust treatment strategy for high-strength landfill leachate at the WAL facility.

## 5. Discussions

### 5.1. Comparative Assessments of WAL Leachate Treatment Scenarios

A comparative assessment of the five (5) appraised simulated treatment scenarios reveals clear differences in technical adequacy, regulatory compliance, and operational suitability for treating the high-strength leachate generated at the WAL. Scenario 1 clearly demonstrates the inadequacy of the existing treatment configuration for managing high-strength landfill leachate. While the system achieves excellent removal of TSSs and phosphorus, its performance for organic matter and nitrogen is fundamentally insufficient. Effluent COD, BOD_5_, ammonia-N, and total nitrogen remain far above NCEC limits, indicating that the biological treatment stages are under-designed for the influent load. The RO unit is forced to operate under excessively high pollutant concentrations, undermining its role as a polishing step. Inferably, Scenario 1 highlights systemic design limitations rather than operational inefficiencies and cannot be considered viable without major structural modification. Meanwhile, Scenario 2 reflects an incremental design approach that fails to adequately address the severity of the influent leachate. Despite the addition of biological contactors, filtration units, and RO, only marginal improvements over the baseline are achieved. Persistent exceedances of BOD_5_, ammonia-N, and total nitrogen indicate that the process train remains poorly matched to the leachate characteristics. The reliance on RO to compensate for insufficient upstream treatment is particularly problematic. Accordingly, Scenario 2 represents a costly upgrade with limited environmental benefit and does not justify implementation given its continued regulatory non-compliance.

The second municipal upgrade with AOP integration in Scenario 3 thus introduces advanced oxidation processes and extended biological treatment, resulting in measurable improvements in pollutant removal. However, despite high percentage removals, absolute effluent concentrations of COD, BOD_5_, ammonia-N, and total nitrogen remain well above regulatory thresholds. This highlights a critical weakness: treatment intensity is insufficient relative to influent strength. While the inclusion of AOP improves robustness and process sophistication, the configuration still relies excessively on downstream polishing without achieving regulatory compliance. Consequently, Scenario 3 represents technical progress but falls short of being a defensible regulatory solution in its current form.

A meaningful shift toward sustainability through the incorporation of AnMBR technology, offering both high solids removal and potential energy recovery, is evident in Scenario 4. Even though the system performed well for TSSs and phosphorus and shows improved nitrogen attenuation, persistent exceedances of COD, BOD_5_, and ammonia-N limit its regulatory acceptability. From a critical standpoint, the scenario’s strength lies in its resource recovery potential rather than compliance performance. Without additional aerobic polishing or intensified post-treatment, Scenario 4 cannot serve as a standalone solution, though it may be valuable as part of a phased or hybrid strategy. Scenario 5 represents the most coherent and technically defensible treatment strategy among the evaluated options. The integration of an anoxic–aerobic MBR with RO substantially improves process control, reduces unit redundancy, and enhances the treatment reliability. Full compliance is achieved for TSSs, total nitrogen, and total phosphorus, while remaining exceedances in BOD_5_ and ammonia-N are comparatively minor and readily addressable through targeted polishing. This scenario offers the best balance between performance, operational simplicity, and scalability, making it the most credible candidate for implementation. Additionally, the Scenario 5 reduces capital redundancy by integrating anoxic–aerobic compartments into a single MBR, lowering infrastructure footprint and civil works costs compared to Scenarios 2–4.

### 5.2. Technological Implications on Sustainable Leachate Treatment

Recent literature reviews have extensively documented the complexity of landfill leachate and the broad spectrum of technologies available for its treatment. In particular, Wang, Z. Qiao et al. [1] emphasized the relevance of conventional and advanced leachate treatment approaches, including biological processes, membrane technologies, and advanced oxidation, and emphasized the need for integrated, multi-stage treatment systems tailored to leachate characteristics and regulatory requirements. Moreover landfill leachate treatment performance is governed not only by removal efficiency but also by leachate maturity, contaminant complexity, cost, and operational sustainability [3,4,31]. Mature leachates typically exhibit COD concentrations of 1000–20,000 mg L^−1^, ammonia-N of 250–4000 mg L^−1^, and very low biodegradability (BOD_5_/COD < 0.1), reflecting dominance of humic substances and refractory nitrogen species [4,32]. The influent used in the present GPS-X^TM^ modeling (COD ≈ 17,050 mg L^−1^; ammonia-N ≈ 989 mg L^−1^) is therefore representative of stabilized landfill leachate reported globally, including arid and developing regions [4].

Recent reviews stress that no single treatment process is sufficient for stabilized landfill leachate and that process sequencing and hybridization largely determine treatment success [1,3,18]. Mature leachates typically exhibit low biodegradability (BOD_5_/COD < 0.1) and high ammonia-N (>500 mg L^−1^), conditions under which standalone biological systems often fail to meet discharge limits even when COD removals exceed 70–80% [2]. This behavior is clearly reproduced in the GPS-X^TM^ results for Scenarios 1–3, where COD removals of ~80–83% still yield effluent COD concentrations > 2900 mg L^−1^, confirming that absolute effluent quality, not removal percentage, governs compliance.

Conventional biological systems are reported to achieve satisfactory performance mainly for young leachates, whereas for mature leachates, COD effluents often remain >1000 mg L^−1^ even at removals exceeding 70% [2,33,34]. This behavior is reproduced in Scenarios 1–3, where GPS-X^TM^ predicts COD concentrations above 2900 mg L^−1^ despite removal efficiencies of ~80%, confirming literature findings that percentage removal metrics alone are insufficient for regulatory compliance

Integrated anaerobic–aerobic systems are widely reported to enhance organic removal, with COD and BOD_5_ reductions of 85–99% under optimized HRTs and long SRTs (>40 d) [35,36]. Scenario 4 (AnMBR + RO) aligns with these observations, achieving ~98% COD removal (COD = 294 mg L^−1^). However, consistent with reports that ammonia persists in mature leachates even after anaerobic treatment (>50 mg L^−1^ in many cases), the modeled ammonia-N (~45 mg L^−1^) remains non-compliant [3,37]. This underscores that anaerobic systems primarily address carbon removal and energy recovery rather than nitrogen compliance.

The literature consistently highlights that combined biological–physicochemical systems outperform single-process configurations. Membrane-based integrated systems, particularly MBR–RO hybrids, are identified in recent reviews as the most reliable option for achieving stringent effluent quality, with RO reported to remove >95–99% of COD, TDSs, ammonia, and persistent organic pollutants when adequately pretreated [3,38,39]. In agreement, the GPS-X^TM^ results for Scenario 5 confirm strong nitrogen control (96% ammonia-N removal and 100% TN removal) and complete solids removal. However, elevated absolute BOD_5_ and COD concentrations persist, reinforcing a key point stressed by previous reported works [3]: under extreme influent loads, even advanced membrane systems may require additional polishing (e.g., adsorption or intensified oxidation) for stabilized leachate.

Modern landfill leachate treatment challenges extend beyond conventional parameters (COD, BOD_5_, NH_3_-N) to include emerging contaminants (ECs) such as pharmaceuticals, endocrine-disrupting compounds, PFAS, and microplastics, typically detected at concentrations of 0.1–300 µg L^−1^ [18]. These compounds are poorly removed by conventional biological systems and often persist through standard treatment trains. While ECs were not explicitly modeled in the present GPS-X^TM^ simulations, the dominance of membrane-based scenarios (Scenarios 4–5) aligns with literature evidence that NF/RO systems can achieve >95–99% rejection of many ECs when adequately pretreated [18]. This strengthens the rationale for selecting membrane-centric configurations for long-term regulatory robustness rather than relying solely on biological performance.

Nitrogen transformation pathways are another critical limitation highlighted in recent works [18]. Recent study indicated that mature leachates commonly contain NH_3_-N concentrations of 400–6000 mg L^−1^, and that anaerobic systems—even when achieving COD removals >85%—often leave ammonia concentrations above 50 mg L^−1^. This trend is reproduced in the present study, where Scenario 4 (AnMBR + RO) achieved ~98% COD removal yet still discharged ~45 mg L^−1^ NH_3_-N. Advanced biological nitrogen removal pathways such as partial nitrification–anammox (PN/A) have been shown to achieve >95–98% inorganic nitrogen removal under optimized conditions [1], suggesting that future upgrades to the modeled systems could incorporate PN/A modules to address ammonia persistence more effectively.

Advanced oxidation processes are frequently reported to remove 80–95% of COD and thereby enhancing biodegradability downstream (BOD_5_/COD ratios from <0.05 to >0.3), when properly dosed [2,40,41]. The marginal improvement observed in the AOP-enhanced GPS-X^TM^ scenario suggests that steady-state modeling may underrepresent refractory organic oxidation, a limitation explicitly noted in recent reviews.

Several research works emphasized the critical role of selection of the technological treatment order. Using physicochemical processes as pretreatment (e.g., coagulation–flocculation or air stripping) can significantly reduce organic and ammonia loading to downstream biological and membrane units, improving overall system robustness [2]. Studies combining air stripping, coagulation, and biological treatment have achieved ammonia removals of 96–98% and COD removals of 90–93% [42]. The absence of a dedicated ammonia-stripping or equivalent pretreatment step in the GPS-X^TM^ scenarios likely explains why ammonia-N remains >40 mg L^−1^ in Scenarios 4 and 5, despite otherwise strong treatment performance.

### 5.3. Economic Implications on Sustainable Leachate Treatment

The techno-economic trends observed in the present WAL leachate treatment assessment are consistent with previously reported cost evaluations of biological and membrane-based wastewater treatment systems. Arif et al. [43] evaluated the economic implications of upgrading conventional activated sludge plants with membrane bioreactor (MBR) technology and reported that MBR systems generally require significantly higher capital investment due to the additional infrastructure associated with membrane modules, pumping systems, and fouling-control aeration. Their analysis showed that the capital cost of MBR systems can be approximately 1.4–1.8 times higher than conventional activated sludge configurations, while treatment costs were in the range of 0.20–0.43 USD m^−3^, depending on process configuration and nitrogen removal requirements. Similar economic patterns are evident in the WAL treatment scenarios evaluated in this study using GPS-X^TM^ process modeling, where treatment trains incorporating membrane systems or additional polishing stages require higher capital investment due to increased equipment complexity, larger reactor volumes, and expanded auxiliary infrastructure.

Comparable conclusions were reported by Latif [44] who conducted a techno-economic comparison of ICEAS, MBBR, and CMAS wastewater treatment configurations using the CAPDETWorks process design and cost-estimation platform. The study estimated construction costs of approximately USD 17–20 million with annual operating costs of USD 1.5–2.1 million yr^−1^, corresponding to treatment costs of 0.39–0.48 USD m^−3^. The results indicated that MBBR systems provided the most favorable economic performance, largely due to lower sludge production and reduced aeration energy demand. Importantly, sludge treatment and handling accounted for approximately 40–59% of total operating costs, highlighting the dominant role of sludge management in determining overall plant economics. Similar cost drivers are identified in the WAL leachate treatment scenarios analyzed in this work, where energy consumption, sludge management, and chemical dosing represent the primary contributors to operational expenditure, particularly for treatment configurations involving intensive aeration or advanced oxidation processes.

Economic analyses of wastewater reuse systems further confirm that treatment complexity and polishing requirements significantly increase lifecycle costs. AbdelMoula et al. [45] reported treatment costs between 0.082 and 0.133 USD m^−3^ for secondary and tertiary municipal wastewater reuse systems, noting that advanced polishing technologies increase both capital and operating expenditures due to higher energy consumption and chemical dosing requirements. Although the absolute treatment costs estimated for WAL leachate (approximately 1.1–5.6 USD m^−3^) are substantially higher due to the extreme pollutant concentrations typical of mature landfill leachate, the underlying economic drivers remain consistent, confirming that energy demand, treatment complexity, and sludge management are the primary determinants of lifecycle cost across different wastewater treatment contexts.

Recent wastewater-to-resource research also highlights opportunities to improve treatment economics through nutrient recovery. Studies on phosphorus recovery systems, such as struvite precipitation technologies, demonstrate that nutrient recovery can reduce chemical consumption for phosphorus removal while simultaneously generating marketable fertilizer products that offset part of the plant operating costs [46]. In addition, phosphorus recovery reduces phosphorus loading in sludge streams, potentially lowering downstream sludge treatment and disposal costs. These findings illustrate the ongoing transition toward resource-recovery-oriented wastewater treatment systems, where pollutant removal is integrated with material and energy recovery to improve lifecycle economic performance. Although nutrient recovery was not explicitly included in the WAL treatment scenarios evaluated in this study, integrating such resource-recovery strategies with biological–membrane treatment configurations represents a promising pathway for further improving the economic sustainability of landfill leachate treatment systems.

### 5.4. Integrated Sustainability Configuration

Against the recent combined-treatment literature, the GPS-X^TM^ simulations reinforce a central conclusion: integrated treatment trains are essential, but their effectiveness depends strongly on pretreatment strategy, process sequencing, and nitrogen management. For highly stabilized leachate, configurations such as MBR + RO represent a robust core treatment, but literature and modeling evidence jointly indicate that ammonia stripping or intensified polishing is often indispensable to achieve full regulatory compliance, particularly under extreme influent conditions typical of arid-region landfills [2].

Beyond treatment efficiency, the recent literature emphasizes that cost and operational trade-offs are decisive factors in differentiating advanced wastewater treatment technologies. Membrane-based systems generally provide superior effluent quality and reliable solid–liquid separation; however, they are often associated with operational challenges such as membrane fouling, concentrate management, and higher operating costs, typically reported in the range of 3–14 USD m^−3^, compared with approximately 1–2.5 USD m^−3^ for conventional biological treatment processes [2,38]. The techno-economic results obtained in the present WAL scenario analysis follow similar trends. Operational expenditures across the evaluated treatment configurations ranged approximately between 1.1 and 5.6 USD m^−3^, with the highest costs associated with the AOP-dominated Scenario 3, reflecting the substantial chemical and energy demand required for advanced oxidation polishing. In contrast, the integrated MBR + RO configuration (Scenario 5) achieved a more balanced cost-performance outcome, with operational costs remaining within 1.3–3.2 USD m^−3^, demonstrating that hybrid biological–membrane systems can maintain high treatment performance while keeping operational expenditures within the lower range reported for advanced treatment systems.

Anaerobic-based systems are often highlighted in the literature for their energy efficiency and potential for biogas recovery, but they frequently exhibit limited nitrogen removal performance, particularly for high-strength ammoniacal wastewaters [37,47]. This limitation is also reflected in the WAL scenario evaluation, where the AnMBR-based Scenario 4, despite offering moderate operational costs (approximately 1.4–3.7 USD m^−3^), showed limited NH_3_-N and TN removal compared with the more integrated treatment configurations. These results reinforce the broader findings reported in the literature that anaerobic processes alone are often insufficient for mature landfill leachate characterized by high ammonia concentrations, requiring additional nitrification–denitrification or polishing processes. Consequently, the selection of compact and modular hybrid systems such as MBR + RO (Scenario 5) represents a balanced approach that combines reliable biological nitrogen removal with membrane-based polishing, while maintaining manageable operational costs, reduced footprint, and improved process controllability. Such integrated configurations are increasingly recommended for mature landfill leachates in high-load or arid environments, where treatment reliability, regulatory compliance, and operational flexibility are critical design considerations.

The recent literature emphasizes that technology maturity and realistic lifecycle costs are critical design factors when selecting advanced wastewater treatment systems. Reported operational expenditures for membrane and advanced oxidation processes (AOPs) are typically in the range of 3–14 USD m^−3^, whereas conventional biological treatment systems generally operate within 1–2.5 USD m^−3^ depending on treatment complexity and sludge management requirements [18]. The techno-economic results obtained in the present WAL analysis show comparable trends. The evaluated treatment scenarios exhibited estimated operational costs of approximately 1.1–5.6 USD m^−3^, with the highest costs associated with the AOP-dominated Scenario 3, reflecting the substantial chemical and energy demand required for advanced oxidation polishing. In contrast, Scenario 5 (integrated MBR + RO configuration) achieved a more balanced cost-performance outcome, with operational costs remaining within the 1.3–3.2 USD m^−3^ range and total lifecycle costs of approximately 2.7–5.5 USD m^−3^ over a 20-year operational horizon.

An important operational consideration associated with membrane-based treatment systems is the management of the reverse osmosis (RO) concentrate stream. Based on the modeled recovery range of 60–80%, approximately 20–40% of the treated leachate volume would be discharged as RO reject, containing concentrated dissolved salts, ammonia, and refractory organic compounds. RO concentrate management remains a critical component of leachate treatment systems and has been widely discussed in previous studies reviewed herein. Several management strategies have been reported for landfill leachate concentrate, including recirculation to the landfill body to enhance waste stabilization, evaporation or solar evaporation ponds, advanced oxidation treatment, or transport to external wastewater treatment facilities. For arid regions such as Saudi Arabia evaporation-based approaches or controlled landfill recirculation may represent practical and economically feasible options for WAL due to high evaporation rates and the existing landfill infrastructure. Recirculation can promote further biodegradation within the landfill mass, while evaporation reduces liquid volume prior to final disposal.

### 5.5. Recommendations for Sustainable Management of WAL Leachate

Based on the integrated technical performance and techno-economic assessment, Scenario 5 (MBR + RO) emerges as the most suitable and sustainable treatment configuration for WAL leachate management. In addition to achieving the highest treatment efficiency and regulatory compliance, this configuration demonstrated balanced lifecycle economics, with estimated operational costs of approximately 1.3–3.2USD m^−3^ and total lifecycle treatment costs of about 2.7–5.5 USD m^−3^ over the 20-year analysis period. Unlike conventional activated sludge scenarios (Scenarios 1–3), scenario 5 requires lower initial capital investment, coupled with reduced sludge production, thus, lower downstream sludge-handling requirements significantly reduce long-term operational burdens and costs. Compared with the AnMBR-based Scenario 4, which offers potential energy recovery but produces ammonia-rich effluent requiring additional polishing, Scenario 5 provides a more balanced solution by simultaneously minimizing sludge generation, simplifying sludge management, and ensuring consistent effluent quality. When treatment efficiency, regulatory compliance, operational reliability, and lifecycle costs are considered together, the MBR + RO configuration represents the most effective pathway toward sustainable landfill leachate treatment at WAL. To achieve better technical performance, sustainability, sludge management, better operational performance and the current global policy targeting a more close-looped wastewater treatment systems [5] the following recommendations are proposed:

- Primary Implementation (MBR + RO)

Scenario 5 should be adopted as the primary treatment configuration because it provides the best balance between treatment efficiency and lifecycle cost. The system consistently meets regulatory limits for TSSs, TN, and TP while maintaining treatment costs within the 1.3–3.2 USD m^−3^ range.

- Optimization of MBR–RO Operation

Maintaining SRT > 20 days, stable dissolved oxygen, and controlled MLSS will ensure reliable nitrification and low sludge production. Proper RO pre-filtration, anti-scalant dosing, and membrane monitoring are essential to reduce fouling, extend membrane lifespan, and control operating costs.

- Targeted Polishing

Additional polishing such as post-aeration, biofiltration, or selective AOP can address the issue of the high efffluent COD and occasional BOD_5_ or ammonia exceedances. These modular units can be intermittently operated to maintain compliance with minimal additional cost.

- Future Role of AnMBR

Scenario 4 (AnMBR) may be considered as a side-stream or future hybrid upgrade for energy recovery. However, due to its limited nitrogen removal, it should not be used as a standalone treatment without additional polishing.

## 6. Conclusions

This study presents a comprehensive, scenario-based evaluation of landfill leachate treatment options for the Wadi Al-Asla landfill in Jeddah, Saudi Arabia, using GPS-X^TM^ process modeling to assess effluent quality, regulatory compliance, and lifecycle economic performance. The characterized leachate exhibited extremely high organic and nitrogenous pollutant loads typical of stabilized, mature landfill leachate, requiring advanced and integrated treatment approaches. Five(5) treatment scenarios combining biological, physicochemical, and advanced treatment processes were systematically evaluated against national discharge standards and sustainability criteria.

Among the assessed configurations, the compartmentalized aerobic-anoxic membrane bioreactor coupled with reverse osmosis (MBR + RO) demonstrated the most robust and balanced performance. The system achieved full compliance for total suspended solids, total nitrogen, and total phosphorus, while significantly reducing COD and ammonia concentrations. In addition to superior treatment efficiency, the configuration showed advantages in sludge minimization, operational stability, and compact footprint, which are critical for sustainable wastewater treatment under extreme loading conditions.

From a techno-economic perspective, the evaluated scenarios exhibited operational costs ranging approximately between 1.1 and 5.6 USD m^−3^ per treated leachate with compartmental aerobic-anoxic MBR + RO configuration maintaining moderate operational costs (1.3–3.2 USD m^−3^) yieding footprint advantage, lower CAPEX and moderate OPEX. The improved solids retention and reduced sludge production associated with the MBR process contribute to lower sludge handling and disposal requirements, partially offsetting membrane-related costs over the system lifecycle. In contrast, advanced oxidation-dominated scenarios showed substantially higher operational costs due to increased chemical and energy demand, while anaerobic-based configurations demonstrated lower energy consumption but limited nitrogen removal performance.

The results further demonstrate that conventional or incrementally upgraded biological systems proposed in municipal treatment strategies, although capable of achieving high percentage removal efficiencies, remain insufficient to consistently meet regulatory discharge limits when treating extremely concentrated landfill leachate. This finding highlights a critical challenge in modern wastewater treatment: moving beyond removal efficiency metrics toward compliance-driven, system-integrated, and economically viable treatment strategies.

The study concludes that the integration of process modeling, regulatory assessment, and techno-economic evaluation provides a more realistic framework for selecting sustainable treatment solutions for high-strength wastewaters. The results support the growing transition toward hybrid biological–membrane systems combined with additional polishing processes as the most effective approach for managing mature landfill leachate. The methodological framework developed in this study is transferable to other high-loaded wastewater supporting the design of resilient, economically sustainable, and compliance-oriented wastewater management systems.

## Figures and Tables

**Table 1:** Different modeled and simulated combination of technologies for WAL leachate.

Scenario	Description	Operational Technologies
Scenario 1	Existing (Baseline)	Chemical treatment + Primary Clarifier + Equalization Tanks + Anoxic Tank + AnMBR + Disinfection + RO
Scenario 2	First Upgrade Proposal by Jeddah Municipality	Anaerobic Digester + Aerobic Tank + Clarifier + Sand Filter + Biological Contactor + Membrane Filter + RO
Scenario 3	Second Upgrade Proposal by Jeddah Municipality	Anoxic/Anaerobic Units + Aerobic Tank + Extended Anoxic Tank + Clarifier + Sand Filter + AOP + Biological Contactor + RO + Unit
Scenario 4	Scenario 1 plus two additional clarifiers	Chemical treatment + Primary Clarifier + Equalization Tanks + Anoxic Tanks + Clarifier 1 + Aeration Tank + Clarifier 2 + AnMBR + RO
Scenario 5	Scenario 4 with Anoxic, Aerobic and clarification combined in three compartments MBR unit	Chemical treatment + Primary Clarifier + Equalization Tanks + three compartments MBR + RO

**Table 2:** Average characteristics of leachate samples of Jeddah Wadi Al-Asla landfill (WAL).

Test Name	Unit	Results	Typical Reported Range [1,2,3,18,30]
Color	-	Black	Dark brown–black
Turbidity	NTU	Highly Turbid	High (site-specific; typically, >100 NTU)
Temperature	°C	24.3	10–40
Total dissolved Solids (TDSs)	mg/L	68,200	2000–60,000
Total Suspended Solids (TSSs)	mg/L	highly Suspended	930–13,333
pH	pH	8	2.69–8.11
Chromium (Cr) (total)	mg/L	0.030135	Trace–0.5
Copper (Cu)	mg/L	0.00325	Trace–1.0
Lead (Pb)	mg/L	0.000915	0.001–5
Nickel (Ni)	mg/L	0.004425	Trace–2.0
Zinc (Zn)	mg/L	0.001155	Trace–5.0
Chloride	mg/L	33,118.19	47–6000
Nitrate (NO_3_)	mg/L	33.833	<1–50
Sulphate	mg/L	1417.14	0.01–3240
Ammonia (NH_3_-N)	mg/L	988.5	400–6000
Nitrogen (total)	mg/L	1222.5	395–5332
Chemical Oxygen Demand (COD)	mg/L	17,050	140–157,200
Biochemical Oxygen Demand (BOD)	mg/L	10,057.5	98–50,000
Toluene	mg/L	0.004484	Trace–3.8
Total Coliform Bacteria	CFU/100 mL	447,500	10^4^–10^7^

**Table 3:** GPS-X model calibration and predictive validation for default scenarios.

Parameter	Calibration			Validation			
	Influent	Measured	GPS-X Predicted	Influent	GPS-X Predicted	Influent	GPS-X Predicted
		Effluent			Effluent		Effluent
COD	27,000	4700	4641	16,900	2910	17,200	2962
BOD	2165	2350	2846	9972	1787	10,143	1818
NH_3_-N	800	300	48.99	1200	76.63	777.00	48.68
Nitrate	33.9	0.37	0.0638	26.2	1.30	41.5	2.064
Total-N	952	672	84.33	1482	107.70	963	76
Total-P	56.6	5.78	6.71	42.5	4.185	42.5	4.26
pH	8.1	7	7	8	7	8.1	7
DO	0.37	2.75	2.775	0.376	2.224	0.321	2.318

**Table 4:** Performances of different WAL leachate treatment scenarios.

Quality Parameter	Influent Concentration mg/L	Scenario 1		Scenario 2		Scenario 3		Scenario 4		Scenario 5		Max Limit NCEC (mg/L)
		Effluent	Removal	Effluent	Removal	Effluent	Removal	Effluent	Removal	Effluent	Removal	
COD	17,050.00	2934.36	82.8	2924.10	82.85	2923.57	82.8	293.52	98.2	2906.48	82.95	120
BOD_5_	8793.38	1801.20	79.5	1790.96	79.63	1790.63	79.6	180.60	97.9	1783.60	79.72	40
TSS	8319.27	0.00	100	0.00	100	0.00	100	0.00	99.9	0.00	100	50
Total Nitrogen	1256.33	92.34	92.6	108.19	91.39	103.78	91.7%	58.3	100%	105	100	15
Ammonia-N	988.50	62.55	93.7%	46.89	95.26	44.23	95.5	45.1	95.43	42.25	95.73	5
Total Phosphorus	38.70	0.0023	99.99%	4.26	88.99	0.00	100%	0.416	98.92	4.189	89.18	30

**Table 5:** Estimated CAPEX and OPEX for the different scenarios (✓ indicates treatment processs involved under each scenario).

Treatment Unit Process	Scenario 1	Scenario 2	Scenario 3	Scenario 4	Scenario 5
Chemical treatment (alum plus lime treatment)	✓			✓	✓
Primary clarifier	✓			✓	✓
Equalization tank	✓			✓	✓
Anoxic tank	✓		✓	✓	
Secondary clarifier		✓	✓	✓	
Anaerobic digester		✓			
Anoxic tank	✓		✓	✓	
Activated sludge tanks	✓	✓	✓	✓	
Secondary clarifier		✓	✓	✓	
Sand filter		✓	✓		
Biological contactor		✓	✓		
AOP			✓		
MBR	✓			✓	✓
Anaerobic MBR					
Membrane filter					
RO unit	✓	✓	✓	✓	✓
Disinfection	✓	✓	✓	✓	✓
CAPEX (USD million)	15–27	11–22	16–32	13–25	9–20
Energy (kWh/m^3^ treated)	1.5–3.5	0.8–2.5	3.0–10	1.5–3.5	1.2–3.0
Energy USD/m^3^ treated	0.6–1.6	0.5–1.3	1.0–2.4	0.6–1.6	0.6–1.4
Chemicals USD/m^3^ treated	0.25–0.8	0.08–0.3	0.3–1.3	0.25–0.6	0.15–0.6
Maintenance USD/m^3^ (incl. sludge management)	0.5–1.3	0.3–1.0	0.5–1.4	0.5–1.3	0.4–1.1
TOTAL OPEX USD/m^3^	1.4–4.0	1.1–3.2	1.9–5.6	1.4–3.7	1.3–3.2
Total Lifetime Cost USD/m^3^ (20 years operations)	3.5–7.0	2.8–5.5	4.5–10	3.2–6.5	2.7–5.5
Relative Footprint	Low–Medium	Medium–High	Medium–High	Low–Medium	Low
Overall Relative Ranking (prioritized: lowest cost + smallest footprint; 1 = best, 5 = worst)	**4** • Moderate cost and footprint • Good effluent quality • Higher sludge and energy than simpler MBR • Not optimal for cost/compactness focus	**2** • Strong lifetime cost savings • Biogas offsets energy and sludge • Larger footprint (conventional bio + digester) • Excellent value if energy recovery utilized	**5** • Highest lifetime cost and OPEX • Very high energy and chemicals (AOP) • Footprint not small enough to offset • Least favorable under cost priority	**3** • Balanced cost and footprint • Reliable MBR + bio removal • Slightly higher cost than Sc5 • Practical mid-tier choice	**1** • Lowest cost (CAPEX and lifetime) • Smallest footprint (compact MBR core) • Efficient sludge and maintenance • Best overall for cost + space savings

**Table 6:** Net present value (NPV) comparison (20 years, 8% discount, 700 m^3^·d^−1^).

Scenario	CAPEX (M USD)	TOTAL OPEX (USD/m^3^)	LCC/NPV Low (M USD)	LCC/NPV Midpoint (M USD)	LCC/NPV High (M USD)
Scenario 1	15–27	1.4–4.0	18.51	27.77	37.03
Scenario 2	11–22	1.1–3.2	13.76	21.89	30.03
Scenario 3	16–32	1.9–5.6	20.77	33.41	46.05
Scenario 4	13–25	1.4–3.7	16.51	25.40	34.28
Scenario 5	9–20	1.3–3.2	12.26	20.14	28.03

## Data Availability

Data are contained within the article or Supplementary Material if any.

## Supplementary Materials

The following supporting information can be downloaded at https://www.mdpi.com/article/10.3390/bioengineering13030359/s1.

## Abbreviations

The following abbreviations are used in this manuscript:

WAL	Wadi Al-Asla Landfill
MBR	Membrane Bioreactor
RO	Reverse Osmosis
AnMBR	Anaerobic Membrane Bioreactor
AOP	Advanced Oxidation Process
COD	Chemical Oxygen Demand
BOD_5_	Biochemical Oxygen Demand (5-day)
TN	Total Nitrogen
NH_3_–N	Ammonia Nitrogen
TSSs	Total Suspended Solids
TP	Total Phosphorus
NOx	Nitrogen Oxides (or Nitrates/Nitrites)
GPS-X^TM^	(Modeling Software Name)
CAPEX	Capital Expenditure
OPEX	Operational Expenditure
SRT	Solids Retention Time
SCADA	Supervisory Control and Data Acquisition
SAR	Saudi Riyal
UF	Ultrafiltration
ZLD	Zero Liquid Discharge
LCC	Lifecycle Cost
PPP	Public-Private Partnership
FO	Forward Osmosis
