# Supplementary material for: Techno-Economic Assessment of Integrated Wastewater Technologies for Sustainable Treatment of Highly Loaded Landfill Leachate Using GPS-XTM
Source: Bioengineering (Basel). 2026 Mar 19;13(3):359. doi: 10.3390/bioengineering13030359 (PMC13024435; doi:10.3390/bioengineering13030359)
Supplement: Supplementary file 1 [file bioengineering-13-00359-s001.zip › bioengineering-4184240-supplementary.pdf]

<u>**Economic Assessment Assumptions and Cost Basis**</u>

The preliminary techno-economic assessment was conducted for a treatment capacity of 700 $m^3 d^{-1}$**,** consistent with the modeled plant capacity. Cost estimation was performed using literature-reported cost coefficients for high-strength landfill leachate treatment systems

Table S1 The Adopted Cost Estimation Ranges based on Literature, US EPA, WEF etc (Bhojwani et al., 2019; de Almeida et al., 2020; Dizayee et al., 2023; Ilyas et al., 2021; Lo et al., 2015; Metcalf & Eddy, 2014; Sharma et al., 2026; US EPA, n.d.; Young et al., 2013)

Name of Unit	CAPEX (USD, approximate range for 700 m³/day scale)	Energy Consumption (USD /m³ treated)	Chemicals (USD/m³ treated)	Maintenance (USD /m³)	TOTAL OPEX (USD/m³ treated, annual basis)
Chemical treatment (Alum plus Lime treatment)	USD 0.5–3 million (precipitation/clarification)	Low (~0.05–0.15)	0.1–0.6 (alum/lime)	0.05–0.2	0.2–1.0
Primary Clarifier	USD 0.3–1.5 million	Very low (<0.05)	Minimal	Low (0.01–0.1)	0.05–0.2
Equalization tank	USD 0.8–3 million (flow balancing)	Low (mixing ~0.05–0.15)	Minimal	Low	0.1–0.3
Anoxic Tank	USD 0.8–3 million (denit.)	Low–medium (0.1–0.3)	Carbon if needed (0.05–0.2)	0.05–0.15	0.2–0.6
Secondary Clarifier	USD 0.5–2 million	Low (<0.05)	Minimal	Low	0.05–0.2
Anaerobic Digester	USD 3–15 million (pretreatment)	Low (biogas offsets some)	Minimal	0.1–0.4	0.2–1.0 (net lower w/ biogas)
Activated sludge tanks	USD 4–12 million (aerobic)	0.3–0.8 (aeration)	Nutrients (0.05–0.2)	0.1–0.3	0.5–1.5
Sand filter	USD 0.8–3 million	Low (0.05–0.15)	Minimal	0.05–0.15	0.1–0.4
Biological Contactor (e.g., RBC)	USD 2–8 million	Medium (0.2–0.5)	Low	0.1–0.3	0.4–1.0
AOP (Advanced Oxidation Processes)	USD 5–20 million (Fenton/ozone/UV)	High (0.5–2.0+)	0.2–1.0 (reagents)	0.2–0.8	1.0–4.0
MBR (Membrane Bioreactor)	USD 8–30 million	0.4–1.5 (aeration + membranes)	Low–medium	0.2–0.7 (cleaning)	0.8–2.5

Anaerobic MBR	USD 10–35 million	Lower (0.3–1.0)	Low	0.2–0.7	0.7–2.0
Membrane Filter (e.g., UF/NF standalone)	USD 4–15 million	0.2–1.0	Low	0.1–0.5	0.5–1.5
RO unit	USD 8–35 million (full-scale/modular)	High (0.8–3.0+ kWh/m³ → 0.08–0.3+ at USD 0.1/kWh)	0.05–0.3 (anti-scalant/acid)	0.2–0.8 (membranes)	0.4–2.0(0.13–0.27 in cases; higher amortized ~USD 8+/m³ long term)
Disinfection	USD 0.8–3 million (UV/chlorine)	Low–medium (0.05–0.2)	0.05–0.2	Low	0.1–0.4

Bhojwani, S., Topolski, K., Mukherjee, R., Sengupta, D., & El-Halwagi, M. M. (2019). Technology review and data analysis for cost assessment of water treatment systems. *Science of The Total Environment*, *651*, 2749–2761. https://doi.org/https://doi.org/10.1016/j.scitotenv.2018.09.363

de Almeida, Ronei, Bila, Daniele Maia, Quintaes, Bianca Ramalho, & Campos, Juacyara Carbonelli. (2020). Cost estimation of landfill leachate treatment by reverse osmosis in a Brazilian landfill. *Waste Management & Research*, *38*(10), 1087–1092. https://doi.org/10.1177/0734242X20928411

Dizayee, K. K. H., Raheem, A. M., & Judd, S. J. (2023). The Cost Benefit of Refinery Effluent Pretreatment Upstream of Membrane Bioreactors. *Membranes*, *13*(8). https://doi.org/10.3390/membranes13080715

Ilyas, M., Kassa, F. M., & Darun, M. R. (2021). Life cycle cost analysis of wastewater treatment: A systematic review of literature. *Journal of Cleaner Production*, *310*, 127549. https://doi.org/https://doi.org/10.1016/j.jclepro.2021.127549

Lo, C. H., McAdam, E., & Judd, S. (2015). The cost of a small membrane bioreactor. *Water Science and Technology*, *72*(10), 1739–1746. https://doi.org/10.2166/wst.2015.394

Metcalf, W., & Eddy, C. (2014). Wastewater Engineering: Treatment and Resource Recovery, Fifth Edition. *Wastewater Engineering: Treatment and Resource Recovery, Fifth Edition*, 1–2018.

Sharma, V., Nagpal, G., Naveen, B. P., & Gupta, P. (2026). An updated review on landfill leachate treatment methods. *Discover Chemistry*, *3*(1), 34. https://doi.org/10.1007/s44371-026-00473-3

US EPA. (n.d.). *Cost Model Documentation*.

Young, T., Smoot, S., Peeters, J., & Cote, P. (2013). When does building an MBR make sense? How variations of local construction and operating cost parameters impact overall project economics. *Proceedings of the Water Environment Federation*, *2013*, 6354–6365. https://doi.org/10.2175/193864713813716444
